# Wearable Systems for Monitoring Mobility-Related Activities in Chronic Disease: A Systematic Review

**DOI:** 10.3390/s101009026

**Published:** 2010-10-08

**Authors:** Lara Allet, Ruud H. Knols, Kei Shirato, Eling D. de Bruin

**Affiliations:** 1 Care Services Directorate, Unit of Physiotherapy Research and Quality Assurance, Geneva University Hospital and University of Geneva, Geneva, Switzerland; 2 University of Applied Sciences of Western Switzerland, HES-SO, Geneva, Switzerland; E-Mail: Lara.Allet@hcuge.ch; 3 Department of Rheumatology and Institute of Physical Medicine, University Hospital Zurich, Zurich, Switzerland; E-Mail: Ruud.Knols@usz.ch; 4 Institute of Human Movement Sciences and Sport, D-Biology, ETH Zurich, Switzerland; E-Mail: hey_soleil@bluewin.ch

**Keywords:** biosensors, movement analysis, mobility, locomotion, rehabilitation, healthcare

## Abstract

The use of wearable motion sensing technology offers important advantages over conventional methods for obtaining measures of physical activity and/or physical functioning in individuals with chronic diseases. This review aims to identify the actual state of applying wearable systems for monitoring mobility-related activity in individuals with chronic disease conditions. In this review we focus on technologies and applications, feasibility and adherence aspects, and clinical relevance of wearable motion sensing technology. PubMed (Medline since 1990), PEdro, and reference lists of all relevant articles were searched. Two authors independently reviewed randomised trials systematically. The quality of selected articles was scored and study results were summarised and discussed. 163 abstracts were considered. After application of inclusion criteria and full text reading, 25 articles were taken into account in a full text review. Twelve of these papers evaluated walking with pedometers, seven used uniaxial accelerometers to assess physical activity, six used multiaxial accelerometers, and two papers used a combination approach of a pedometer and a multiaxial accelerometer for obtaining overall activity and energy expenditure measures. Seven studies mentioned feasibility and/or adherence aspects. The number of studies that use movement sensors for monitoring of activity patterns in chronic disease (postural transitions, time spent in certain positions or activities) is nonexistent on the RCT level of study design. Although feasible methods for monitoring human mobility are available, evidence-based clinical applications of these methods in individuals with chronic diseases are in need of further development.

## 1. Introduction

Chronic diseases are leading causes of death and disability in both developed and developing countries [1,2]. Chronic disease conditions and diseases that directly influence the components of fitness and performance are related to perceived health among middle-aged and older adults [3–5]. Inactivity is at the origin of several chronic diseases [6], and regular physical activity substantially modifies the development and progression of most chronic degenerative disease states [7]. Thus, it is not surprising that exercise is considered to play a critical role in the management of chronic illnesses [8,9]. Avoidance of a sedentary lifestyle by engaging in at least some daily physical activity is a prudent recommendation for reducing the risk of developing chronic diseases and postponing premature mortality at any age [7].

Because functional status is an important factor affecting quality of life and healthcare utilization [10], valid outcome measures of physical activity and/or physical functioning are of utmost importance. Especially an understanding of the quantity and quality of physical (in)activity is particularly important in addressing rehabilitation needs of the individual with a chronic disease.

Several tools are available for measuring physical activity (PA). PA can be measured by the use of questionnaires, interviews or a diary. However, these methods are mostly retrospective and contain a subjective judgement which leads to a certain amount of imprecision causing limited reliability [11]. Objective, performance-based measures of physical function have the disadvantage that performance at the time of assessment may not represent the usual performance of the tested individual [12]. The alternative to assess outcomes through proxy is only recommended for individuals who cannot be evaluated with other methods. This method also seems to have limited reliability and validity [13] and seems unsuitable for longer time periods of measurement [14].

A recent development is the use of wearable motion sensing technology for studying human movement. Based on the use of miniaturised motion sensors, methods are available for long-term monitoring of daily PA and the assessment of motor functioning under real-life conditions [15]. These methods are relevant for studying motor functioning in older people [11], but as yet it is unclear to what extent and with what effect such methods have been applied in individuals with chronic disease conditions. The variety of studies, providing a wealth of experimental data, makes it difficult to get a clear view of the clinical relevance of these methods for the assessment of physical functioning. An overview of application methods in patients suffering from chronic diseases is currently lacking.

In the present study, we review and evaluate the application of wearable motion sensing technology for monitoring mobility-related activity in individuals with chronic diseases. We used the International Classification of Functioning, Disability, and Health (ICF) as a reference to organize the results and to limit the scope of this study [16]. Mobility in this context is changing and maintaining body position (ICF d410-d429) or walking and moving (ICF d450-d469) [17]. The aim is to identify the clinical relevance and feasibility of applying wearable motion sensing technology in these individuals and to reveal existing problems that have to be solved. In particular we aimed to clarify the following aspects: (a) to assess the quality and internal validity of studies using body-fixed sensors for measuring daily activity in patients with chronic diseases, (b) to provide an overview of ambulatory activity measurement tools used for chronic diseases and their properties, (c) to summarize the mode of application of different body-fixed sensors which measure individuals’ daily activity, (d) to sum up encountered problems and technical failures, (e) to assess user acceptance, and (f) to conclude about the clinical relevance.

## 2. Methods

Because of the large variety of chronic diseases, it was important to focus on some of the most frequently occurring chronic diseases for this review. Among the main chronic diseases [18] we selected Osteoarthritis, Cardiovascular Diseases (CVD), Typ-2-Diabetes mellitus and Chronic Obstructive Pulmonary disease (COPD) for this review. With these four types of chronic diseases three main domains of physical impairment that are influenced by these chronic illnesses can be covered: physiological problems (CVD&COPD), metabolic syndromes (Diabetes) and physical impairments (Osteoarthritis). Of all global deaths in 2005, 39% were because of these chronic diseases [1].

In March 2009 a librarian performed an electronic search on PubMed and the Physiotherapy Evidence Database (PEDro). The PubMed search strategy included the following keywords: (*chronic diseases OR cardiovascular disease OR CVK OR diabetes OR diabetic OR COPD OR osteoarthritis*) *AND* (*gait disorder OR walking OR kinematic OR gait analysis system OR gait analysis device*) *AND* (acceleromet* OR pedomet* OR gyrosco* OR wearable system OR activity monitor OR motion sensing OR instrumentation OR equipment) *AND* (*physical activity OR motor activity OR activity*). Because the PEDro database does not have a MeSH key terms registry, this search strategy was modified for this database.

The search included all articles between January 1990 and March 2009 which were published in English or German. We only included articles with a randomized controlled trial design. To identify further studies, reference lists of primary articles were reviewed.

### 2.1. First Selection Based on Abstracts

Based on the title and abstract a first selection was performed. Papers were included in the full text review when they satisfied the following inclusion criteria: (1) *Types of studies*: only randomized controlled trials; (2) *Types of participants*: patients in the studies had one of the following diseases: Diabetes, Osteoarthritis, COPD or CVD; (3) *Measurement tool:* only trials evaluating mobility characteristics of individuals with wearable motion sensing technology that is intended for long-term monitoring under free living conditions were included (“wearable system”, “Accelerometer”, “Pedometer” or “activity monitor”). Laboratory-based analysis systems were excluded; *Types of outcome measures*: ICF categories “changing and maintaining body position” or “walking and moving” had to be reported; *Intervention*: an intervention focussing on mobility/physical activity must be reported.

Disagreement regarding inclusion of the studies was resolved by consensus between authors (RHK/KS). In case of disagreement between the two reviewers, a third person (EDB) decided whether the article should be included in the systematic review.

### 2.2. Method for Quality Assessment in Selected Full Text Articles

Quality assessment of the included articles was based on the PEDro scale [19,20] and independently assessed by two reviewers (KS&RHK or EDB). The PEDro scale is based on the Delphi list developed by Verhagen *et al.* [21], which is a set of 11 criteria for quality assessment: (1) eligibility; (2) use of randomization; (3) concealment of treatment allocation; (4) equivalence (or similarity) of groups at baseline; (5) blinding of the subjects; (6) blinding of the therapists; (7) blinding of the outcome assessors; (8) intention-to-treat analyses; (9) reporting of point estimates; (10) measures of variability of the primary outcome and (11) adequacy of follow-up and use of between-group statistical comparisons [22].

Every full text article was read by two independent reviewers (KS, EdB and RHK) and judged with the quality scale. Percentage agreement between raters and Cohen’s kappa were calculated with GRAPHPAD software (Version 2002–2005; GRAPHPAD Software Inc, San Diego, Ca), and were interpreted in accordance with Landis and Koch’s benchmarks for assessing the agreement between raters: poor (<0), slight (0.0 to 0.20), fair (0.21 to 0.40), moderate (0.41 to 0.60), substantial (0.61 to 0.80), and almost perfect (0.81 to 1.0) [23]. Disagreement regarding inclusion of the studies was resolved by consensus between authors (RK, EDB, KS). As recommended by van Tulder *et al.* [24] a third reviewer (LA) was used in the event of any disagreement between the two reviewers regarding the methodological quality of a trial. The PRISMA-statement was followed for reporting items of this systematic review and meta-analyses [25].

## 3. Results and Discussion

**Figure 1.**
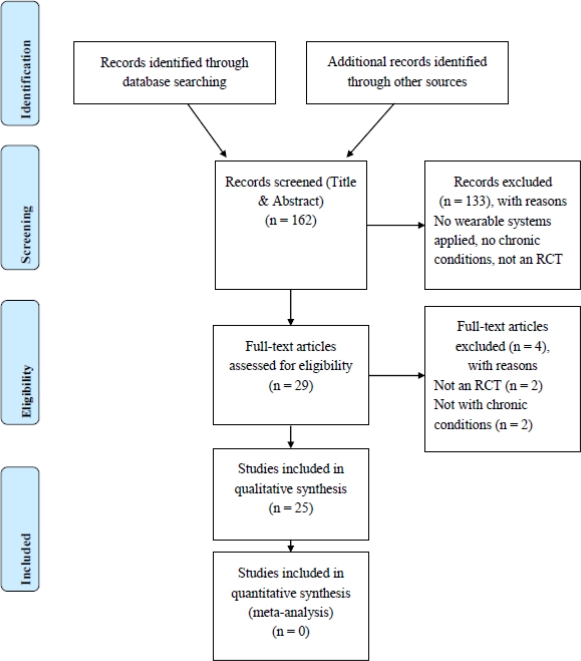
Flow diagram (*Adapted from:* Moher, D., Liberati, A., Tetzlaff, J., Altman, D.G., The PRISMA Group (2009). Preferred Reporting Items for Systematic Reviews and Meta-Analyses: The PRISMA Statement. PLoS Med 6(6): e1000097. doi:10.1371/journal.pmed1000097).

***First Selection Based on Abstracts***: 162 abstracts were identified with the search strategy. The title and abstract reading excluded 133 studies (Figure 1). None of the articles had to be excluded on the basis of a language problem. During the full text reading four further articles had to be excluded. Finally, 25 articles could be included in the quality judgement; two RCTs focussing on arthritis [26,27], four focussing on COPD [28–31], six focussing on CVD [32–37], and 13 focussing on Diabetes [38–50].

The three articles written by Kirk *et al.* [44,45,47] were all based on the same study and, therefore, treated as one publication. Although these three papers were focussing on different research questions, they essentially used the same set of data for the parameters of interest for this review. Table 1 gives an overview of all included studies with their corresponding PEDro-scores.

***Data Quality***: The quality scores of studies ranged from 3 to 9 points out of a maximum of 11 points. The mean quality score was 6.3 ± 0.3 points. Both the median and mode values were 6 points (Table 1). The Kappa value was 0.91 (with 95%-confidence interval ranging between 0.87–0.96), indicating an agreement between raters considered to be ‘almost perfect’.

### 3.1. Technologies and Applications

There were 15 studies that evaluated walking using a pedometer (step counters). Six studies used uniaxial accelerometers for activity measurements (e.g., by calculating the integral over the rectified accelerometer signal over a certain duration) to obtain overall measures of physical activity level and/or estimations of energy expenditure or step counts. Five studies used multiaxial movement sensors for long-term monitoring of activity patterns. No studies used methodologies (e.g., gyroscopes) to characterise either spatio-temporal parameters during long periods of walking or to evaluate characteristics of postural transitions or used combinations of uniaxial devices for posture detection. Two studies used a combination of a pedometer and an accelerometer for the determination of energy expenditure. The sum of reported methods is larger than 25.

Three different applications could be identified: in 21 studies the wearable device was used to measure walking activity of patients. The wearable systems were also used as a study outcome measure (the number of steps, movements (accelerations of the body) or energy consumption at baseline and follow up were reported and compared). Pedometers were further used to encourage patients increasing their physical activity-level (five studies) or for controlling their activity (three studies); see Section 3.4 for more details. For this purpose the wearable devices were used as a direct feedback, e.g., the device indicated the number of steps per day performed. Table 2 summarises the various wearable sensors with their properties and applications. Different instruments for measuring physical activity were used across the studies. The systems uncovered and their general characteristics are summarised in Table 2.

### 3.2. Mode of Application

Table 2 contains an overview of the types of devices that have been used with their properties and applications. In most studies the choice of instrument was either a pedometer or an accelerometer. Two studies used the combination of an accelerometer and a pedometer [26,48]. The majority of studies used devices that are attached at hip level. Two studies apply devices that should be attached to the ankle.

### 3.3. Feasibility and Adherence

Six studies reported difficulties and study limitations with wearable activity measurement tools. Three studies reported on adherence problems to wear the devices or to wear the devices in the proper measurement position [27,42,48]. One study suspected that differences between study groups became not apparent because of the high signal-to-noise-ratio of the devices used [31]. One study reported inconsistencies between self-report measures of physical activity and accelerometers. The inconsistencies were presumed to be caused by the wearable system [33]. One study remarked on the lack of being able to conclude about the intensities of the physical activities performed during the study period [34]. Table 3 gives an overview of the problems and limitations mentioned. None of the studies reported about user acceptance of the measurement tool.

### 3.4. Clinical Relevance

Completed studies where individuals or groups were followed up in time and where the effects of interventions on physical activity were investigated totalled 21 studies: two completed RCTs focussing on arthritis [26,27], four completed RCTs focussing on COPD [28–31], five RCTs focussing on CVD [32–34,36,37], and ten completed studies focussing on Diabetes [38–49] (where [44,45,47] were treated as being one study). The remaining studies were either published study protocols or presenting base line data only.

Preferred reporting items [25] were derived from each included trial on: (1) characteristics of trial participants and the trial’s inclusion criteria; (2) type and aim of intervention; (3) type of mobility outcome measure (including the level of change in mobility outcome (using wearable systems); (4) conclusions from the studies. Because the interventions and reported outcome measures varied markedly, we focused on describing the studies and their results, and on qualitative synthesis rather than meta-analysis.

#### 3.4.1. Characteristics of Osteoarthritis Studies

Both osteoarthritis studies were randomised controlled trials published in English. The duration of the Talbot *et al.* study [26] was 24 weeks; 12 weeks for the intervention and an additional 12 weeks follow-up period. Toda *et al.* [27] report a 6-week study duration. The two included studies involved 74 participants with a mean age (SD) of 66.7 (SD 8.2) years. The main inclusion criteria entailed being aged 60 and older, pain in one or both knees on most days, difficulty performing at least one functional task because of pain, radiographic evidence of osteoarthritis (OA) [26], as well as adult obese women with a body mass index (BMI) >26.4 and a diagnosis of mild OA. One trial was conducted in the USA [26] and the other one in Japan [27].

The intervention received in Talbot *et al*. was 12 hours of the Arthritis Self-Management program over 12 weeks (received by both the control and intervention group). The intervention group received additional individualized instruction in the use of a pedometer, with the goal of increasing the step count by 30% of the baseline step count. The participants wore the three-axial Tritrac-R3D accelerometer and a non-sealed DigiWalker pedometer for 72 consecutive hours. The participants were given a physical activity goal and were receiving feedback about their physical activity through the instruction to record the daily steps taken at the end of each day of the measurement period [26]. Toda *et al.* [27] applied a weight control program in both groups. Group A received an appetite suppressant once per day plus a NSAID taken orally twice a day for 6 weeks (n = 22). At both breakfast and lunch 150 mL low calorie soup with essential nutrients were taken. Group B (n = 18) received the same NSAID for 6 weeks. Participants in both groups were instructed to walk continuously for up to 30 minutes each day. The participants wore a sealed pedometer (Seiko, Tokyo, Japan) for six weeks during working hours and leisure time. Results, recorded as steps per day, were checked in the clinic every two weeks.

The primary outcome assessed was change in daily steps walked from baseline [26] and values of weight, BMI, percent body fat, change score, and metabolic correlates of obesity [27]. The effects of the physical activity interventions on daily walking yielded statistically significant results for change in daily step counts [26]. In this study the daily steps walked showed a significant group by-time interaction after controlling for age. From baseline to completion of training, a 23% increase in daily steps occurred in the intervention group and a 15% decrease in the control group. The participants in the intervention (walking exercise) group increased their pedometer steps/week on an average by 818 steps per day, compared to minus 680 steps/day in the self-management group (p = 0.04) [26]. Toda *et al.* claim to have effectively increased the number of steps taken per day in both groups, as compared to values of a reference population. This was a secondary outcome of their study and this claim was not, however, substantiated with data [27].

#### 3.4.2. Characteristics of CVD Studies

All five CVD studies were randomised controlled trials published in English. The duration of the studies was 12 weeks [32], 24 weeks [34], 26 weeks [36], 6 months [37], and 12 months [33], respectively. These five studies involved 272 participants with a mean age of 58.7 (SD 5.5) years. The main inclusion criteria entailed male non-smokers, with hypercholesterolemia [32], postmenopausal women with borderline to stage 1 hypertension [34], adult African Americans with newly diagnosed hypertension [36], and patients with a clinical diagnosis of chronic heart failure [33,37].

The interventions received were the request to walk briskly for at least 30 minutes on at least five out of every seven days for a period of 12 weeks. In each exercise bout, energy expenditure was measured using a Caltrac accelerometer. Energy expenditure, duration of bout and rate of perceived exertion (RPE) were recorded in an activity log book. Intervention participants were trained to walk at an intensity equivalent of RPE of 12–14, which is equivalent to an energy expenditure of 5–7.5 kcal per minute. Control participants were requested to maintain their current activities of daily living. The aim was to investigate whether a home-based physical activity program meeting current guidelines was effective in improving the lipid profile in hypercholesterolaemic men [32]. Hughes and colleagues applied 11 weeks of supervised exercise (1-hour sessions, twice a week), medical evaluation, education and psychological support. The experimental group received an exercise consultation after baseline and 6-month assessments and a support phone call three months after each consultation. Control participants received a phone call on topics unrelated to exercise three and nine months after the baseline assessment. Outcome measures recorded at baseline were repeated at 6 and 12 months. The aim was to compare the long-term effect of the exercise consultation with standard exercise information on maintenance of physical activity and cardio-respiratory fitness [33]. Fifteen women in the exercise group of Moreau and co-authors walked three km·d^−1^ above their daily lifestyle walking, whereas nine women in the control group did not change their activity. Walking activity was self-measured with a pedometer in both groups. Women in the EX group were provided with a target number of steps that would lead to a 3-km increase in daily walking. The target steps were added onto their baseline step value in order to prevent a decline in their current daily lifestyle activity. Women in the CON group were asked not to change daily activity and subsequently wore a pedometer for one week each month to document their walking. [34]. The intervention group of the Sohn *et al.* study was encouraged to walk an extra 30 minutes per day, five to seven days a week. The control group was not given this advice and received standard medical care. The researchers wanted to determine whether the encouragement of walking an extra 30 minutes a day decreases blood pressure in adult African Americans with newly diagnosed hypertension [36]. Witham and colleagues divided their exercise intervention into supervised and home phases. In the supervised phase (0 to 3 months), participants attended exercise classes as outpatients in groups of three to four, twice a week during the first three months. Between 17 and 20 sessions were offered during the 3-month period. Participants in the control group received usual care. The aim was to determine whether a seated exercise program designed specifically for older frail patients with heart failure (HF) would be well attended and would lead to improvements in exercise capacity, everyday activity, and health status [37].

Physical activity was measured using the Caltrac accelerometer set to display physical activity energy expenditure (kcal). Participants recorded wear time, total activity energy expenditure and any bouts of moderate activity ≥30 minutes or vigorous activity of ≥20 minutes each day in a diary. Accelerometers were worn by all participants for one week prior to randomization to establish baseline physical activity and throughout the 12-week experimental period [32]. Participants wore the uniaxial MTI accelerometer model 7164 (MTI, Shalimar, Florida, USA) on their right ankle during all waking hours for seven days, except during water activities. Accelerometers were set to record activity in 1-minute epochs. Activity counts/min recorded over the seven monitored days were summed to produce total activity counts/week [33]. Subjects were given a Yamax SW-200 pedometer (Yamax, Inc., Tokyo, Japan) to wear throughout the day for a 1- to 2-week period before beginning the 24-week walking program in order to document pre-intervention daily lifestyle walking activity. An average daily value was computed and served as the baseline value. Stride length was measured of each subject and walking distance was calculated [34]. All subjects in the Sohn *et al.* study used a Yamax SW-200 pedometer (Yamax, Inc., Tokyo, Japan) to record the number of steps walked on a daily basis [36]. Daily activity over a 7-day period was measured using the Stayhealthy RT3 triaxial accelerometer (Stayhealthy Inc, Monrovia, California). The device was mounted anteriorly on the participant’s waistband and recorded summed acceleration counts at 1-minute intervals [37].

Participants in the intervention group performed 97.6% of the frequency of walking requested and expended 120% of the intensity of walking requested. They used a mean of 1,675 kcal per week carrying out the walking program, but showed a reduction of 12% (p < 0.01) in net energy expenditure at the follow-up. The control group showed a non-significant reduction in net energy expenditure of 5% (p = 0.08), compared to baseline values [32]. Total activity counts/week measured by accelerometry did not change significantly from baseline to 6 months (98%) and 12 months (98%) in the experimental group. In the control group, total activity counts/week decreased non-significantly from baseline to 6 and 12 months by 5.2% and 8%, respectively [33] At baseline, women in the EX and CON groups walked an average of 5,400 ± 500 and 7,200 ± 700 steps·d^−1^, respectively, equivalent to walking 3.4 ± 0.3 and 4.7 ± 0.4 km·d^−1^ (significantly different between EX and CON groups, *p* < 0.05). Women in the EX group increased their daily walking by 4,300 steps (2.9 ± 0.2 km·d^−1^; significantly different from baseline and from the CON group, *p* < 0.05) and averaged a total of 9,700 ± 400 steps·d^−1^ (including baseline steps) across the 24-week walking program (significantly different *vs*. the CON group). The women in the CON group did not change their walking activity over 24 weeks (−0.3 ± 0.3 km·d^−1^). In this study, pedometers were used to help the women achieve their walking prescription and it was documented that women were increasing walking activity from their usual daily lifestyle walking with this approach [34]. Sohn *et al.* report that both the intervention and control group increase their daily steps, however, they do not report data that substantiate this observation. The authors assume that an explanation of this finding lies in the fact that in both groups the pedometer acted inadvertently as a motivator for walking [36]. The most important finding in the Witham study was the significant increase in everyday physical activity measured by accelerometry in the exercise group at six months. The increase in everyday activity was most likely to have resulted from behavioral changes brought about by participation in the program, telephone follow-up, keeping a diary, and the setting of walking targets [37].

#### 3.4.3. Characteristics of type 2 Diabetes Mellitus Studies

All type 2 Diabetes mellitus studies were randomised controlled trials published in English: three trials from the UK [43–47], three trials from the USA [38,42,48], two from Norway [39,40], one from Australia [41], and one from Canada [49]. The duration of the studies was 5 weeks [46], 6 weeks [38], 12 weeks [39], 24 weeks [49], 6 months [40,41,44,45,47], and 12 months [42,43,48], respectively. These ten studies involved 720 participants with a mean age of 57.3 (SD 8.5) years. The main disease related inclusion criteria entailed type 2 diabetes [38,40,42–47], type 2 diabetes and overweight [39], type 2 diabetes, overweight/obese and sedentary subjects [41,49], and people with diabetic peripheral neuropathy [48].

Kirk *et al.* (2001) applied an exercise consultation and standard exercise information (experimental) or standard exercise information alone (control). The aim was to evaluate the effect of exercise consultation on promotion of physical activity in people with type 2 diabetes. The outcomes were stage of exercise behaviour, physical activity levels (7-day recall questionnaire & accelerometer), and quality of life. The intervention did not include a physical activity target and no feedback about physical activity was provided to the participants from the accelerometer [46]. Araiza and colleagues (2006) randomized patients into two groups after 10 days of baseline activity. The control group (n = 15) was instructed to continue with their baseline activity for six weeks. The active group (n = 15) was instructed to walk at least 10,000 steps per day, five or more days per week, for six weeks. The authors wanted to determine whether a recommendation to walk 10,000 steps per day would result in significant improvements in glycemic control, insulin sensitivity, and cardiovascular risk in patients with type 2 Diabetes mellitus. Subjects in the active group received feedback from a pedometer [38]. In the study of Bjorgaas *et al.* (2005) participants in the exercise group were offered a 12-week program of 2 × 1.5-h supervised exercise per day. This program did not include a specific physical activity target. The control group was not given any specific recommendations concerning physical activity. The aim was to investigate the relationship between pedometer-registered activity, aerobic capacity (VO2max) and self-reported activity and fitness. Pedometers were given to the patients with the instruction to wear the devices during three consecutive weekdays [39]. In the UK-based intervention that resulted in three different papers [44,45,47] the focus of the intervention was exercise consultation, based on the transtheoretical model, combined motivational theory and cognitive behavioral strategies into an individualized intervention to promote physical activity. The aim of the consultation was to encourage participants to accumulate 30 minutes of moderate physical activity most days of the week. There was no target goal set for physical activity besides the time limit. The aim of this project was to evaluate the long-term (6 and 12 months) effectiveness of physical activity counseling in people with type 2 diabetes [44,45,47]. Bjorgaas and co-workers (2008) wanted to investigate whether the use of a pedometer increases walking and/or enhances beneficial outcomes in an intervention study aimed at physical activity. Study participants were allocated to a pedometer or no pedometer group. All were encouraged to increase walking. Subjects in the pedometer group registered pedometer steps at three days, twice per month for six months, and were encouraged to increase their daily step count based on the mean number of steps during month 1. Participants who did not use a pedometer were encouraged to increase the average daily time spent walking guided by a logbook. There were no fixed target goals set for participants [40]. To investigate the impact of using a pedometer on time spent walking in a coaching intervention was the aim of Engel and her group. For that purpose participants were allocated to either a pedometer and coaching (intervention) group or a coaching-only (control) group. Coaching for both groups involved education, goal setting, and supportive/motivational strategies to increase time spent walking. The goal setting to achieve the physical activity goals differed between the groups: the pedometer group set goals based on the number of steps per day, while the other group set goals based on a certain amount of time spent walking each day [41]. The study of Tudor-Locke *et al.* (2004) examined the effectiveness of the First Step Program (FSP). In addition to the primary outcome of physical activity (measured as steps/day), the authors also examined whether increased physical activity was related to improvements in cardiovascular health, glycemic control and lipid profiles. During an adoption phase (initial four weeks), participants were asked to attend four weekly group meetings (individual attendance was recorded). They were given pedometers and the program manual containing goal setting and problem-solving exercises, as well as calendars for self-monitoring steps/day. No specific advice was given concerning diet or glycemic control. During the adherence phase (subsequent 12 weeks), participants were asked to use their pedometers (as a feedback tool) and calendars for goal-setting and self-monitoring. The control group was individuals on a waiting list [49]. To determine the effect of a lower extremity exercise and walking intervention program on weight-bearing activity and foot ulcer incidence in people with Diabetes Mellitus (DM) and Peripheral Neuropathy (PN) was the aim of another study. Intervention components included leg strengthening and balance exercises; a graduated, self-monitored walking program (part 1); and motivational telephone calls every two weeks (part 2). Participants were encouraged to increase activity by adding a minimum of 100 steps to their daily activities every two weeks. They self-monitored walking with an Accusplit Eagle 170 pedometer as a self-motivating means. The control group did not receive guidance to undertake walking. Both groups in this study received diabetic foot care education, regular foot care, and eight sessions with a physical therapist. [48]. Keyserling and co-workers (2002) wanted to determine whether a culturally appropriate clinic- and community-based intervention for African-American women will increase moderate-intensity physical activity. Three treatment conditions were: clinic and community (group A), clinic only (group B), or minimal intervention (group C). The clinic-based intervention (groups A and B) consisted of four monthly visits with a nutritionist who provided counseling to enhance PA and dietary intake that was tailored to baseline practices and attitudes; the community-based intervention (group A) consisted of three group sessions and 12 monthly phone calls from a peer counselor and was designed to provide social support and reinforce behavior change goals; and the minimal intervention (group C) consisted of educational pamphlets mailed to participants. Participants were encouraged to increase the “minutes” (duration) of moderate or vigorous activities or to add intensity to mild/sedentary activities [42]. Participants were randomized to receive a physical activity consultation delivered by a person or in written form to increase physical activity levels over 6 and 12 months in a UK-based study. The aim was to compare the effectiveness of two methods of physical activity promotion for people with type 2 diabetes, in contrast to standard care. The intervention part did not include physical activity target formulation or goal setting [43].

Kirk *et al.* (2001) assessed physical activity with a uniaxial accelerometer (CSA Inc; no further specifications) that delivered activity counts at one-minute intervals for a seven-day period. Upon return, activity monitors were downloaded and a total weekly physical activity count was calculated for each participant [46]. In the Araiza *et al.* study each subject wore a Yamax Digiwalker step counter (SW-701, New Lifestyles, Kansas City, MI) throughout the day, except for sleeping and bathing, and was trained regarding proper placement and use of the pedometer. Each morning the pedometer was reset to zero, and each evening the subject recorded the steps accumulated during the day in an activity log [38]. Bjorgaas *et al.* (2005) gave their participants a pedometer (Yamax Digi-Walker ML AW-320, Yamax, Tokyo, Japan) and these were instructed to wear it clipped to their clothing or belt at the waist, centred over the foot. Each participant registered his ambulatory activity by pedometer during three consecutive weekdays. Participants were instructed to set the pedometer to zero at the beginning of the first day and to record the number of steps each evening. All participants completed the registration satisfactorily [39]. The primary outcome measure in the three studies from the Kirk group [44,45,47] was measurement of bodily acceleration from a CSA uniaxial accelerometer (Computer Science and Applications, Shalimar, FL). Monitors were worn for seven days during waking hours. Monitors were downloaded on return and a total weekly activity count was calculated. Thus, patients did not receive direct feedback from the monitors about their physical activity. Bjorgaas *et al.* (2008) used a Yamax Digi-Walker three weekdays twice per month to register ambulatory activity [40]. Changes in cardio respiratory fitness and anthropometric measurements were used to corroborate changes in physical activity. A Yamax-Digi-Walker was used to measure steps per day in the intervention group of Engel *et al.* Participants were instructed to reset the pedometer to zero each morning before positioning it on their waistband [41]. Tudor-Locke and colleagues assessed physical activity (defined as steps/day) with a Yamax SW-200 pedometer over three consecutive days while engaging in usual activities. Each person was instructed to set the pedometer to zero the first morning, and then seal the cover with a sticker, thus preventing feedback from the pedometer during the measurement phase [49]. Physical activity as a primary outcome was assessed with a StepWatch accelerometer. Changes in total daily steps, steps taken in 30-minute exercise bouts, and minutes per week were assessed [48]. Keyserling *et al.* assessed physical activity by Caltrac accelerometer to objectively measure physical activity energy expenditure. There were no devices used to give feedback on physical activity [42]. The primary outcome of the King *et al.* (2009) study was physical activity derived from the ActiGraph GT1M accelerometer. This device delivered weekly step counts and body accelerations per minute and were worn for seven days [43]. The device was not used as a feedback or motivational tool.

Physical activity counts/week increased by 4% in the experimental group and decreased by 9% in controls. A significant difference was recorded for the change in activity counts per week from baseline to follow-up between the experimental and control group [46]. The main finding of Araiza’s group was that subjects in the active group significantly increased physical activity by 69% during the intervention phase of the study, whereas there was no change in the physical activity of the control group. The authors attributed this increase to the use of the pedometer [38]. Bjorgaas and colleagues (2005) observed that both exercise and control groups tended to increase pedometer activity [39]. The experimental group in the study of Kirk *et al*. had increased levels of physical activity from baseline to six months, with no decrease from 6 to 12 months. In the control group, accelerometer counts per week decreased from baseline to 12 months. Between-group differences were recorded in physical activity (recall and accelerometer) at 12 months. Experimental participants significantly increased total activity. Control participants recorded no significant change [44,45,47]. In the pedometer group of Bjorgaas *et al.* (2008), the number of steps per day did not increase from month 1 to month 6. The authors concluded that the use of a pedometer did not increase walking or enhance beneficial metabolic outcomes. They suspect that an increase in step count may be attributed to regular counselling and goal setting, and not to the use of a pedometer *per se* [40]. Engel *et al*. (2006) observed with an ANCOVA approach that group membership had no influence on the time spent walking. Sedentary older adults were able to achieve the physical activity targets with a coaching intervention equally well as with an added pedometer. The authors acknowledge, however, that the pedometer group focussed on steps per day, and thus they also included steps made during incidental bouts of activity in their analysis, whereas the coaching group focused on time spent walking [41]. Intervention (with step goal) participants increased their physical activity by approximately 3,000 steps/day from baseline (p < 0.01), which was a significant improvement relative to the waiting list controls (p < 0.0001). At 24 weeks follow-up the difference between groups was no longer significant [49]. At 6 months, bout-related daily steps increased 14% from baseline in the intervention group and decreased 6% from baseline in the control group. Although the groups did not differ statistically in the change in total daily steps, steps had decreased by 13% in the control group at 12 months [48]. For the primary study outcome, the comparison of PA levels during 0.5 and 1 year of follow-up, there was a significant difference between group A und C after 12 months and between B and C following six months. One limitation mentioned is a possible bias in PA measurement, which may have resulted from differences in actual Caltrac wearing time by treatment group [42]. Kirk *et al.* (2009) observed no significant interaction between time and group in accelerometer counts, step counts, and time in moderate intensity or above, over 6 and 12 months. There was a significant main effect of time on accelerometer counts (P < 0.001), step counts (P = 0.005), time in moderate intensity or above (P = 0.006) and the 7-day recall (P = 0.007), with an overall decrease between baseline and 12 months (P < 0.01) and 6 and 12 months (P < 0.01) across all measures of physical activity. Neither a theory-driven physical activity consultation delivered by a person nor in written form were better than a standard care leaflet at increasing physical activity levels over 6 and 12 months in the full study group [43].

#### 3.4.4. Characteristics of COPD Studies

The four COPD studies were randomised controlled trials published in English; two trials were conducted in the USA [28,31], one in the UK [30], and one in the Netherlands [29]. The duration of the studies was 7 weeks [30], 8 weeks [28], 9 weeks [29] and one year [31], respectively. These four studies involved 331 participants with a mean age of 66.9 (SD 9.3) years. The main disease-related inclusion criteria entailed diagnosis of COPD [28–30] or self-reported diminished physical functioning related to a pulmonary problem or pulmonary function impairment [31].

Experimental group subjects were instructed to walk at their own pace for 20 to 45 min, two to five times a week, using distractive auditory stimuli (DAS) with a portable audiocassette player in the Bauldoff *et al.* study [28]. The control group received the same instructions, but no DAS. All subjects recorded the distance and time walked using self-report (pedometers and daily logs). The aim was to determine if DAS in the form of music would promote adherence to a walking regimen following completion of a pulmonary rehabilitation program (PRP) and, thereby, maintenance of gains achieved during the program. Primary outcome measures were perceived dyspnea during activities of daily living (ADL) and 6-min walk (6 MW) distance [28]. De Blok *et al.* instructed their experimental group to follow a regular rehabilitation program plus an additional lifestyle physical activity counseling intervention. They also received direct feedback from a pedometer. The control group followed a regular COPD rehabilitation program only. In the control group, patients wore the pedometer 1 week prior to the rehabilitation and 1 week during week 9 of their rehabilitation. Patients in the experimental group wore the pedometer for 10 weeks (1 week prior to rehabilitation and 9 weeks during rehabilitation). Patients were instructed to wear the pedometer over the course of the entire day until going to bed, and to record daily step counts in a diary. Patients also recorded in their diary at which time they put the pedometer on and at which time they put the pedometer off. [29]. The aim of this intervention was to stimulate patients to increase their daily physical activity level by incorporating lifestyle physical activities into daily life. Patients received exercise counseling in order to motivate them to enhance physical activity in daily life and wore a pedometer in order to monitor changes in physical activity behavior. The pedometer was used as a motivational and feedback tool [29]. Sewell *et al*. randomized patients to a conventional 7-week general exercise program or an individually targeted exercise program. Daily activity was measured using ambulatory activity monitors. This group aimed to establish whether pulmonary rehabilitation (PR) improves domestic function and daily activity levels in COPD and whether individually targeted exercise is more effective than general exercise. The authors did not formulate specific physical activity goals [30]. Steele and co-workers applied a 12-week adherence intervention (weekly phone calls and home visit) including counseling on establishing, monitoring, and problem-solving in maintaining a home exercise program. The authors wanted to evaluate the effectiveness of an exercise adherence intervention to maintain daily activity, adherence to exercise, and exercise capacity over one year after completion of an outpatient pulmonary rehabilitation program. The control group consisted of continuing care with the referring provider and individual recommendations for continuation of the exercise program. Primary outcomes were daily activity, self-reported daily exercise, and functional capacity. The experimental group received a digital pedometer (no details specified) for self-monitoring and an exercise handbook describing aspects of the program. There was no goal setting for the physical activity and there was no feedback about physical activity from the accelerometer [31].

Following baseline testing, all subjects were given and instructed in use of an electronic pedometer (Model 342; Sportline; Campbell, CA), which measured walk distance to 1/100 of a mile precision [28]. In addition, all subjects were instructed in completion of a log, which included date, amount of time, and distance walked as measured by the electronic pedometer. There were no specified target goals, e.g., increase of walking, for the participants. The pedometer was rather used as a recording device and not as a feedback tool [28]. The cumulative distance walked by the DAS group was 19.1 ± 16.7 miles compared to 15.4 ± 8.0 miles for the control group, a 24% difference. Despite this difference, self-report exercise log data were similar for the two groups [28]. The primary outcome measure was daily physical activity and was measured by the Yamax Digi-Walker SW-200 (Tokyo, Japan). Mean steps/day was evaluated and patients were asked to set a goal for seeking their maximal physical activity limit once (measured in numbers of steps). The fourth counseling session was carried out in week 7 of the rehabilitation and dealt with consolidation of physical activity behavior. Patients were asked to set a goal for their personal physical activity norm, which should be between their mean steps/day until then and their maximal number of steps [29]. The overall amount of daily activity was measured as primary outcome by Sewell *et al.* using a uniaxial ambulatory activity monitor (Z80—32k V1 Int; Gaehwiler Electronics; Hombrechtikon, Switzerland). All of the patients wore the monitor on two consecutive days for a period of 12 hours on each day. Patients did not receive feedback from the device and it was not used as a motivational tool [30]. Physical activity when awake was measured as body movement in vector magnitude units (VMU) using the RT3 accelerometer on four 6-day measurements at home. An average of the daily activity in VMU performed over the six days was computed [31].

Subjects who used DAS while walking had improved functional performance and decreased perceptions of dyspnea [28]. The compliance of recording daily step counts in the dairy turned out to be very high; 91.7% during pretest and posttest in experimental and control group and 95.5% during the 10 weeks that the experimental group wore the pedometer. There were no significant differences in the mean time of wearing the pedometer during the day between experimental group (13.1 hour/day) and control group (13.8 hour/day). The experimental group increased their mean steps/day by 1,430 steps/day (+69%) for six days without rehabilitation, whereas the control group showed an increase of 455 steps/day (+19%). The difference in pretest and posttest between the experimental and control groups was clinically relevant. De Blok *et al.* study showed that the number of steps/day increased after nine weeks of rehabilitation. The additional lifestyle physical activity counseling program with feedback of a pedometer showed a clinically relevant increase in steps/day [29]. Statistically significant percentage improvements were noted in both training groups. An increase in activity monitor counts of 29.18% was demonstrated by the GEP and of 40.63% by the ITEP. The authors acknowledge that a possible weakness of the study was that the goal direction may not have been sufficiently explicit in order to affect any change in the identified functional goals of the subjects. They also remark that the monitors utilized in this study only record the quantity of daily activity, and they do not provide any information relating to individual domestic task completion [30]. Steele *et al.* observed that no statistically significant difference between the intervention and control groups of daily activity at completion of the adherence intervention (short term) existed (p > 0.20). Neither were there significant differences between the intervention and control groups after one year. The authors remarked that a factor that may explain the lack of differences between the intervention and control groups in the accelerometer measure of daily activity was the finding that the RT3 data had a high signal-to-noise ratio that swamped any differences in daily activity [31].

### 3.5. Discussion

Our quality evaluation of included papers yielded good to excellent correspondence between reviewers. In general, the scores ranged from low to high. This can be explained by the fact that four of the included papers were ongoing studies that presented baseline data only. There were ten randomised studies with a score above six points. The articles from the Kirk *et al.* group that were considered as being one study inconsistently reported the Pedro quality items. This resulted in different Pedro scores. The item “blinding” certainly negatively influenced the appraisal of the study quality. None of the articles fulfilled the criteria about blinding, an issue known to be quite impracticable in most clinical intervention studies.

Pedometers or accelerometers were used for three different applications: (a) to measure the individuals’ activity behaviour, (b) to encourage subjects to increase their activity-level or (c) to control individuals’ activity level by using a direct feedback. In order to assess individuals’ activity level authors either assessed the subjects’ covered walking distance, the number of steps, or the energy consumption. The results of our review indicate that the used devices were suitable for verifying whether someone achieves his/her personal physical activity goal. This was especially the case in studies that used devices that allowed a direct feedback on performance. However, the use of pedometers, with the aim to encourage people to increase their activity and compare these to control groups, seems questionable. Our findings show conflicting results in this regard. The devices used are not always able to differentiate between exercise and control groups when only subtle differences between groups exist. This can, of course, partly be explained by the lack of credibility of the interventions applied. However, some authors explicitly state that they think their findings are in part attributable to the disappointing measurement characteristics of the accelerometer used [31]. Bravata *et al.* evaluated the association of pedometer use with increasing physical activity, and they suggest that the use of a pedometer is associated with significant increases in physical activity and significant decreases in body mass index and blood pressure [51]. Our findings add to this knowledge that the feasibility of wearable motion sensing technology also seems to relate to the reliability of the used devices in unsupervised measurement settings with the various studied individuals. However, there are also studies that find that subjects who used a pedometer did not increase walking during the study and health benefits were not enhanced by the use of a pedometer, e.g., Bjorgaas *et al.* (2008) [40]. This finding seems to contrast with studies that showed that the use of a pedometer leads to an increase in step counts [38,49]. The difference between these studies might be attributed to the inclusion of regular counselling and goal setting that leads to an increase in step count and not to the use of a pedometer per se. This assumption is in line with the results of a review on physical activity interventions to improve walking in cancer survivors [52]. The review concluded that goal setting combined with feedback from a pedometer and counselling are the likely factors that explain increases in physical activity. There is, thus, increasing evidence that the definition of a step goal may be the key motivational factor in increasing physical activity [51]. Together the results of these studies suggest that daily walking activity is likely to improve only when a realistic step goal is defined.

Not all devices have the same accuracy for all different application fields. The Yamax Pedometer for example, a device currently used in clinical trials, seems to enable precise and reliable data [53,54]. However, Crouter *et al.* [55] showed that the spring-levered pedometer (Yamax Digiwalker SW-200) became less accurate with increasing BMI, increasing waist circumference, and greater pedometer tilt, whereas a piezoelectric pedometer (New Lifestyles NL-2000) was not affected by these variables. They thus concluded that in overweight and obese individuals, a piezoelectric pedometer is more accurate than a spring-levered pedometer especially at slower walking speeds [55]. Such a finding may guide the selection of a wearable system for certain chronic diseases that often present themselves with concurrent overweight of subjects, e.g., diabetes mellitus. However, no relationship between the type of the chronic disease and the type of the assessment tool could be identified.

The number of reported problems or technical failures is small. This may indicate that body-fixed sensors for measuring daily activity in form of pedometers and accelerometers are good and reliable measurement instruments. The devices seem to be technically mature. Pedometers afford an inexpensive objective measure of physical activity. They are normally worn at the belt line and are sensitive to vertical movement [56]. Pedometers are designed to detect the total number of steps taken, distance travelled and energy expenditure. In most of these electronic devices, a horizontal spring-suspended lever arm deflects during movement with the acceleration of the hips [56]. The main limitation of waist-attached pedometers is, however, that the accuracy at slow walking speeds is reduced. The vertical accelerations of the waist are less pronounced during slow walking and cause an inaccuracy in measuring the steps [53,57,58]. In addition, pedometers are not able to assess intensity, frequency and duration of activity [57]. The majority of the studies in this review did not consider these limitations.

Most commercially available accelerometers are, as this review also reflected, waist-worn devices that measure body movements in terms of acceleration [57,59]. The majority of accelerometer sensors are sensitive to movement in the vertical plane, but some of them detect accelerations also in the antero-posterior and/or medio-lateral planes [60]. In contrast to pedometers, accelerometers are capable of describing intensity, frequency and duration of physical activity [57]. The major drawback of such waist-fixed accelerometers is that they seem to be affected by multiple factors such as movement style, gait speed, and mode and location of attachment [57,59,61,62]—a finding that potentially has implications for applications in individuals with chronic diseases. To overcome the limitations of the previous waist-attached devices, two-dimensional microprocessor-based accelerometers (e.g., the Step Activity Monitor, SAM) were developed [59,62] and used in two studies reviewed [48,63]. The SAM is an ankle-worn instrument and designed to detect movements of the lower extremity. It can objectively measure the amount, the pattern and also the intensity of activity and has adaptable sensitivity settings. The step activity monitor is able to record different activities such as walking, running, stair use, and bicycling [64]. Additionally, human steps can be detected by SAM for a wide variety of gait styles and walking pace [11,62].

All studies included in this review assess mobility as walking and moving (ICF d450-d469). RCTs that assess mobility as changing and maintaining body position (ICF d410-d429) in individuals with chronic diseases were not identified. There were for example no studies that used wearable systems to characterise either spatio-temporal parameters during long periods of walking or those that evaluated characteristics of postural transitions. Neither were combinations of uniaxial devices for posture detection used. This finding has meaning in relation to an additional limitation of most commercially available activity monitors: they are not able to measure static movements such as standing and sitting. New emerging activity monitors, for example Physilog [65] and ActivPAL [60], have been developed with the aim of providing information about body postures. Physilog and ActivPAL can divide human activity into lying, sitting, standing and walking. ActivPAL furthermore offers information on step count and metabolic equivalents to each activity [66,67]. The observation seems justified that more research is necessary in individuals with chronic conditions to determine whether postural detection systems are an appropriate instrument for long-term measuring of physical activity in such individuals. Especially an understanding of the quantity and quality of physical (in)activity seems particularly important in addressing rehabilitation needs [65], e.g., for assessment and intervention procedures.

The focus on mobility that we had—we limited the scope on “changing and maintaining body position (ICF d410-d429)” or “walking and moving (ICF d450-d469)” [17]—can be considered as a limitation of our review. Additional functions and sensations of the cardiovascular and respiratory system (ICF b450-b469) are measurable with wearable systems as well, e.g., with an ActiHeart device [68–70]. Information from this or from comparable systems, where activity and heart rate are measured next to calculations of energy expenditure for ambulatory activities, provides additional means of PA assessments. Such additional information has relevance for individuals with chronic diseases. It might well be that focusing on these additional functions and sensations of the cardiovascular and respiratory system (ICF) might have led to additional relevant studies.

The patients suffering from one of the selected chronic diseases do generally not need a permanent stationary treatment. These patients live at home and perform their usual daily occupation. Thus, it is important to assess their activity level in their living environment. Wearable technology allows clinicians to gather data where it matters the most to answer this question, *i.e.*, the home and community settings. A wearable motion sensing system is likely to be operationally feasible if it meets the ‘needs’ and expectations of both the developer and user, where user can be either defined as study participant or clinical professional assessing subjects. User acceptance is thus an important determinant of operational feasibility [11]. This review could not exhaustively describe the technical failures and individuals’ acceptance as initially intended. The included studies did not extensively report on this issue. It is unlikely, however, that all patients readily accept a wearable system. The user issues identified include forgetting to put the device on, complying with the intended wearing time, and adhering to the application protocol of the device. Future studies should also include information on these aspects of wearable devices.

Finally it has to be noticed that a particularly high number of articles considering patients with diabetes (10 completed studies and one ongoing study) and patients with CVD (6 articles; 5 completed, 1 ongoing project) could be identified. Only few articles considered patients with COPD or osteoarthritis. It may be that the high prevalence of patients with diabetes influenced this result. Furthermore, the importance of regular physical activity is particularly recommended in patients with metabolic diseases. There is uncertainty, however, as to what the best treatment for osteoarthritis is. A critical appraisal shows that the overall quality of existing treatment guidelines is sub-optimal, and consensus recommendations are not always supported by the best available evidence [71]. Although there is outstanding evidence for the benefit of exercise therapy in knee osteoarthritis, there is at the same time some indication that exercise is underused as a treatment modality for this chronic condition [72]. The findings of this review seem to substantiate this indication.

### 3.6. Other Reviews

There are several other reviews that explore the potential of wearable motion sensing technology. The majority of these reviews are, however, non-systematic and narrative in nature and often confined to accelerometers only [73,74], focussing on a specific disease [75] or on the (older) adult population at large [11,76–78]. One systematic review investigates the validity aspects of using pedometers in investigating physical activity [79], one systematic review is focussing on COPD patients [80], and one systematic review investigates the use of pedometers to increase physical activity in adult outpatients [51].

## 4. Conclusions

The number of studies that use pedometers (step counters) and/or accelerometers to obtain overall measures of physical activity level, or estimations of energy expenditure, form the majority when subjects with chronic disease conditions are studied. In contrast, the number of studies that use movement sensors for long-term monitoring of activity patterns, such as postural transitions or time spent in certain positions or activities, is nonexistent on the RCT level of study design.

Identified feasibility and adherence aspects of wearable motion sensing technology mainly relate to the signal-to-noise-ratio of some devices in unsupervised measurement settings and (to the) compliance to the application protocol of the devices by the various studied individuals. Information about the user acceptance when attached to certain body parts or clothing, which impedes measurements, is not available from the studied RCTs.

Although feasible methods are available for monitoring activity patterns and posture detection, these methods have not yet been applied on a large scale in individuals with chronic conditions. Evidence-based clinical applications of these methods (e.g., for fall prediction and outcome assessment) are in need of (further) development. At present, therefore, the use of wearable systems for monitoring movement activities in people with chronic conditions has not yet reached its full potential. Our findings indicate that the challenge for the various developed wearable motion sensing systems is evaluation in “real life” settings.

Before selecting a wearable motion sensing technology clinical practitioners should judge the psychometric properties of the desired device, estimate the feasibility of using the technology for the intended population, and formulate clear goals of measuring physical activity in clinical settings in order to receive credible information.

## Figures and Tables

**Table 1.** Overview of studies.

**Author**	**PEDRo Quality score**
***Osteoarthritis***	

Talbot *et al*., 2003 [26]	6
Toda *et al*., 1998 [27]	6

***COPD***	

Bauldoff *et al*., 2002 [28]	6
de Blok *et al*., 2005 [29]	6
Sewell *et al*., 2005 [30]	6
Steele *et al*., 2008 [31]	7

***CVD***	

Coghill *et al*., 2008 [32]	8
Hughes *et al*., 2007 [33]	7
Moreau *et al*., 2001 [34]	7
Sohn *et al*., 2007 [36]	5
Witham *et al*., 2005 [37]	8

***Diabetes mellitus***	

Araiza *et al*., 2006 [38]	6
Bjorgaas *et al*., 2005 [39]	6
Bjorgaas *et al*., 2008 [40]	5
Engel *et al*., 2006 [41]	6
Keyserling *et al*., 2002 [42]	8
LeMaster *et al.*, 2008 [48]	9
Tudor-Locke *et al*., 2004 [49]	5
Kirk *et al.*, 2001 [46]	7
Kirk *et al*. 2003, 2004 [44,45,47]	7/6/8
Kirk *et al*., 2009 [43]	9
Yates *et al*., 2008 [50]	4

**Table 2.** Body fixed sensors with their properties and applications.

***Device type***	***Outcome Measure(s)/Placement***
***Pedometers***	

Yamax Digi-walker Modell SW-200 [26,29,34,36,49,50], SW 700 [41], ML AW-320 [40], (Yamax, Tokyo, Japan), SW 701 [38] (New Lifestyles, Kansas City, MI)	Step counts, distance, Energy Expenditure/Waist
Sportline Distance Pedometer Model 342 (Sportline, Campbell CA) [28]	Distance/Waist
Pedometer (Seiko, Tokyo, Japan) (no further specifications) [27]	Step Counts/Waist
Accusplit Eagle 170 (Pleasanton, CA) [48]	Step counts, distance, Energy Expenditure/Waist
NL-800 (New Lifestyles, USA)	Step Counts/Waist

***Uniaxial Accelerometers***	

Z80/32KV1 activity monitor (Gaehwiler Electronics; Hombrechtikon, Switzerland) [30]	Activity counts/Waist
Caltrac Accelerometer (Muscle Dynamics, Torrance, CA, USA)	Energy Expenditure/Waist
MIT Accelerometer Modell 7164 (MIT, Shalimar, Florida, USA) [33]	Activity counts/Ankle
Computer Science and Applications (CSA) uniaxial Accelerometer, (Computer Science and Applications, Shalimar, Florida, USA) [44,45,47]	Activity counts/Waist

***Multiaxial Accelerometers***	

Step Activity Monitor (SAM) (Prosthetic Research Study, Seattle, WA, USA)/StepWatch Activity Monitor (OrthoCare Innovations, Washington DC) [48,63]	Step counts, step rate/Ankle
RT3 Accelerometer (Stayhealthy Inc, Monrovia, CA, USA) [31,37]	Activity counts, vector magnitude, energy expenditure/Waist
ActiGraph model GT1M (ActiGraph LLC, Pensacola, FL, USA)[43]; Manufacturing Technology, Fort Walton Beach, FL) [35]	Step counts, activity counts, energy expenditure/Wrist, waist, ankle
Tritrac-R3D (Hemokinetics, Madison, WI, USA)[26]	Activity counts, vector magnitude, energy expenditure/Waist

**Table 3.** Reported encountered problems and technical failures.

***Author***	***Type/Brand***	***Problems reported***
Toda *et al*., 1998 [27]	Pedometer (Seiko, Tokyo, Japan) (no further specifications)	Three of 18 participants in the control group (17%; see Table 1) had forgotten to attach pedometers for several days and were excluded from the study. The remainder of this group (n = 15) were evaluated [27].
Steele *et al*., 2008 [31]	RT3 Accelerometer (Stayhealthy Inc., Monrovia, CA, USA)	The RT3 data had a high signal-to-noise ratio that swamped any differences in daily activity between the groups. This finding was evidenced by large day-to-day variations in VMU (Table 2), further contributing to low power of the RT3 measurement of daily activity. The authors suspect that an arduous side-by-side comparison of the performance characteristics of the TriTrac-R3D and the RT3 might have shown that the TriTrac-R3D had a better signal-to-noise ratio, which would help explain why this device was able to detect subtle differences in daily activity in people who were exercising for greater durations. This was not done, because preventive maintenance and recalibration of the TriTrac-R3D units in possession of the authors were no longer available, and the TriTrac-R3D could no longer be purchased [31].
Keyserling *et al.*, 2002 [42]	Caltrac Accelerometer (Muscle Dynamics, Torrance, CA, USA)	One limitation mentioned is possible bias in PA measurement, which may have resulted from differences in actual Caltrac wearing time by treatment group. This might indicate possible problems with compliance wearing the device. There were, however, no differences in reported wearing time between treatment groups. It is possible that the actual PA energy expenditure was underestimated by the Caltrac, since it does not detect non-ambulatory PA (e.g., arm swinging). However, this bias is consistent for all subjects and is a limitation in the use of vertically oriented accelerometers as a direct measure of PA [42].
Hughes *et al.*, 2007 [33]	Uniaxial MIT accelerometer, Modell GT1M (Manufacturing Technology, Fort Walton Beach, FL)	The decline in total activity counts/week measured by accelerometers did not parallel the marked decrease in self-reported physical activity in the controls. The authors speculate that this discrepancy may be due to limitations of accelerometers since these devices cannot record water activities, activities that increase energy expenditure without a proportional increase in bodily acceleration (e.g., walking uphill) and those requiring a large amount of upper body movement (e.g., washing windows) [33]. The decrease in self-reported activity in the controls could reflect a decline in these activities, which would not be detected by accelerometers.
Moreau *et al*., 2001 [34]	Yamax Digi-Walker, SW-200 (Yamax, Tokyo, Japan)	The authors were unable to determine the intensity of the amount of daily walking that the women included in the study performed [34].
LeMaster *et al*., 2008 [48]	StepWatch (OrthoCare Innovations, Washington)	With respect to protocol adherence there were some participants that attached the StepWatch in the reverse direction causing loss of physical activity data [48].

## References

[1] Beaglehole R, Ebrahim S, Reddy S, Voute J, Leeder S (2007). Prevention of Chronic Diseases: A Call to Action. Lancet.

[2] Yach D, Hawkes C, Gould CL, Hofman KJ (2004). The Global Burden of Chronic Diseases: Overcoming Impediments to Prevention and Control. JAMA.

[3] Johnson RJ, Wolinsky FD (1993). The Structure of Health Status Among Older Adults: Disease, Disability, Functional Limitation, and Perceived Health. J. Health Soc. Behav.

[4] Malmberg J, Miilunpalo S, Pasanen M, Vuori I, Oja P (2005). Characteristics of Leisure Time Physical Activity Associated with Risk of Decline in Perceived Health—a 10-year Follow-up of Middle-Aged and Elderly Men and Women. Prev. Med.

[5] Malmberg JJ, Miilunpalo SI, Vuori IM, Pasanen ME, Oja P, Haapanen-Niemi NA (2002). A Health-Related Fitness and Functional Performance Test Battery for Middle-Aged and Older Adults: Feasibility and Health-Related Content Validity. Arch. Phys. Med. Rehabil.

[6] Katzmarzyk PT, Janssen I (2004). The Economic Costs Associated with Physical Inactivity and Obesity in Canada: an Update. Can. J. Appl. Physiol.

[7] Chodzko-Zajko WJ, Proctor DN, Fiatarone Singh MA, Minson CT, Nigg CR, Salem GJ, Skinner JS (2009). American College of Sports Medicine Position Stand. Exercise and Physical Activity for Older Adults. Med. Sci. Sport. Exerc.

[8] Stuart M, Chard S, Benvenuti F, Steinwachs S (2009). Community Exercise: A Vital Component to Healthy Aging. Healthc. Pap.

[9] (2002). Physical Activity Fundamental to Preventing Disease. Services.

[10] Ferrucci L, Baldasseroni S, Bandinelli S, de Alfieri W, Cartei A, Calvani D, Baldini A, Masotti G, Marchionni N (2000). Disease Severity and Health-Related Quality of Life Across Different Chronic Conditions. J. Am. Geriatr. Soc.

[11] de Bruin ED, Hartmann A, Uebelhart D, Murer K, Zijlstra W (2008). Wearable Systems for Monitoring Mobility-Related Activities in Older People: A Systematic Review. Clin. Rehabil.

[12] Myers AM, Holliday PJ, Harvey KA, Hutchinson KS (1993). Functional Performance Measures: Are They Superior to Self-Assessments?. J. Gerontol.

[13] Magaziner J, Zimmerman SI, Gruber-Baldini AL, Hebel JR, Fox KM (1997). Proxy Reporting in Five Areas of Functional Status. Comparison with Self-Reports and Observations of Performance. Am. J. Epidemiol.

[14] Ferrucci L, Guralnik JM, Studenski S, Fried LP, Cutler GB, Walston JD (2004). Designing Randomized, Controlled Trials Aimed at Preventing or Delaying Functional Decline and Disability in Frail, Older Persons: a Consensus Report. J. Am. Geriatr. Soc.

[15] Zijlstra W, Aminian K (2007). Mobility Assessment in Older People: New Possibilities and Challenges. Eur. J. Ageing.

[16] Dahl TH (2002). International Classification of Functioning, Disability and Health: An Introduction and Discussion of its Potential Impact on Rehabilitation Services and Research. J. Rehabil. Med.

[17] (2001). International Classification of Functioning, Disability and Health (ICF).

[18] (2005). Preventing Chronic Diseases: A Vital Investment.

[19] Giglia E (2008). PEDro: This Well-Known, Unknown. Physiotherapy Evidence Database. Eur. J. Phys. Rehabil. Med.

[20] Maher CG, Sherrington C, Herbert RD, Moseley AM, Elkins M (2003). Reliability of the PEDro Scale for Rating Quality of Randomized Controlled Trials. Phys. Ther.

[21] Verhagen AP, de Vet HC, de Bie RA, Kessels AG, Boers M, Bouter LM, Knipschild PG (1998). The Delphi List: A Criteria List for Quality Assessment of Randomized Clinical Trials for Conducting Systematic Reviews Developed by Delphi Consensus. J. Clin. Epidemiol.

[22] PEDro Physiotherapy Evidence Database.

[23] Landis JR, Koch GG (1977). An Application of Hierarchical Kappa-Type Statistics in the Assessment of Majority Agreement among Multiple Observers. Biometrics.

[24] van Tulder MW, Assendelft WJ, Koes BW, Bouter LM (1997). Method Guidelines for Systematic Reviews in the Cochrane Collaboration Back Review Group for Spinal Disorders. Spine (Phila Pa 1976).

[25] Moher D, Liberati A, Tetzlaff J, Altman DG (2009). Preferred Reporting Items for Systematic Reviews and Meta-Analyses: The PRISMA Statement. PLoS Med.

[26] Talbot LA, Gaines JM, Huynh TN, Metter EJ (2003). A Home-Based Pedometer-Driven Walking Program to Increase Physical Activity in Older Adults with Osteoarthritis of the Knee: A Preliminary Study. J. Am. Geriatr. Soc.

[27] Toda Y, Toda T, Takemura S, Wada T, Morimoto T, Ogawa R (1998). Change in Body Fat, but not Body Weight or Metabolic Correlates of Obesity, is Related to Symptomatic Relief of Obese Patients with Knee Osteoarthritis after a Weight Control Program. J. Rheumatol.

[28] Bauldoff GS, Hoffman LA, Zullo TG, Sciurba FC (2002). Exercise Maintenance Following Pulmonary Rehabilitation: Effect of Distractive Stimuli. Chest.

[29] de Blok BM, de Greef MH, ten Hacken NH, Sprenger SR, Postema K, Wempe JB (2006). The Effects of a Lifestyle Physical Activity Counseling Program with Feedback of a Pedometer during Pulmonary Rehabilitation in Patients with COPD: A Pilot Study. Patient Educ. Couns.

[30] Sewell L, Singh SJ, Williams JE, Collier R, Morgan MD (2005). Can Individualized Rehabilitation Improve Functional Independence in Dlderly Patients with COPD?. Chest.

[31] Steele BG, Belza B, Cain KC, Coppersmith J, Lakshminarayan S, Howard J, Haselkorn JK (2008). A Randomized Clinical Trial of an Activity and Exercise Adherence Intervention in Chronic Pulmonary Disease. Arch. Phys. Med. Rehabil.

[32] Coghill N, Cooper AR (2008). The Effect of a Home-Based Walking Program on Risk Factors for Coronary Heart Disease in Hypercholesterolaemic Men. A Randomized Controlled Trial. Prev. Med.

[33] Hughes AR, Mutrie N, Macintyre PD (2007). Effect of an Exercise Consultation on Maintenance of Physical Activity after Completion of Phase III Exercise-Based Cardiac Rehabilitation. Eur. J. Cardiovasc. Prev. Rehabil.

[34] Moreau KL, Degarmo R, Langley J, McMahon C, Howley ET, Bassett DR, Thompson DL (2001). Increasing Daily Walking Lowers Blood Pressure in Postmenopausal Women. Med. Sci. Sport. Exerc.

[35] Price HC, Tucker L, Griffin SJ, Holman RR (2008). The Impact of Individualised Cardiovascular Disease (CVD) Risk Estimates and Lifestyle Advice on Physical Activity in Individuals at High Risk of CVD: A Pilot 2 × 2 Factorial Understanding Risk Trial. Cardiovasc Diabetol.

[36] Sohn AJ, Hasnain M, Sinacore JM (2007). Impact of Exercise (walking) on Blood Pressure Levels in African American Adults with Newly Diagnosed Hypertension. Ethn. Dis.

[37] Witham MD, Gray JM, Argo IS, Johnston DW, Struthers AD, McMurdo ME (2005). Effect of a Seated Exercise Program to Improve Physical Function and Health Status in Frail Patients ≥70 Years of Age with Heart Failure. Am. J. Cardiol.

[38] Araiza P, Hewes H, Gashetewa C, Vella CA, Burge MR (2006). Efficacy of a Pedometer-Based Physical Activity Program on Parameters of Diabetes Control in Type 2 Diabetes Mellitus. Metabolism.

[39] Bjorgaas M, Vik JT, Saeterhaug A, Langlo L, Sakshaug T, Mohus RM, Grill V (2005). Relationship between Pedometer-Registered Activity, Aerobic Capacity and Self-Reported Activity and Fitness in Patients with Type 2 Diabetes. Diabetes Obes. Metab.

[40] Bjorgaas MR, Vik JT, Stolen T, Lydersen S, Grill V (2008). Regular Use of Pedometer does not Enhance Beneficial Outcomes in a Physical Activity Intervention Study in Type 2 Diabetes Mellitus. Metabolism.

[41] Engel L, Lindner H (2006). Impact of Using a Pedometer on Time Spent Walking in Older Adults with Type 2 Diabetes. Diabetes Educ.

[42] Keyserling TC, Samuel-Hodge CD, Ammerman AS, Ainsworth BE, Elasy TA, Henriquez-Roldan CF, Skelly AH, Johnston LF, Bangdiwala SI (2002). A Randomized Trial of an Intervention to Improve Self-Care Behaviors of African-American Women with Type 2 Diabetes: Impact on Physical Activity. Diabetes Care.

[43] Kirk A, Barnett J, Leese G, Mutrie N (2009). A Randomized Trial Investigating the 12-month Changes in Physical Activity and Health Outcomes Following a Physical Activity Consultation Delivered by a Person or in Written Form in Type 2 Diabetes: Time2Act. Diabetic Med.

[44] Kirk A, Mutrie N, MacIntyre P, Fisher M (2003). Increasing Physical Activity in People with Type 2 Diabetes. Diabetes Care.

[45] Kirk A, Mutrie N, MacIntyre P, Fisher M (2004). Effects of a 12-month Physical Activity Counselling Intervention on Glycaemic Control and on the Status of Cardiovascular Risk Factors in People with Type 2 Diabetes. Diabetologia.

[46] Kirk AF, Higgins LA, Hughes AR, Fisher BM, Mutrie N, Hillis S, MacIntyre PD (2001). A Randomized, Controlled Trial to Study the Effect of Exercise Consultation on the Promotion of Physical Activity in People with Type 2 Diabetes: A Pilot Study. Diabetic Med.

[47] Kirk AF, Mutrie N, Macintyre PD, Fisher MB (2004). Promoting and Maintaining Physical Activity in People with Type 2 Diabetes. Am. J. Prev. Med.

[48] Lemaster JW, Mueller MJ, Reiber GE, Mehr DR, Madsen RW, Conn VS (2008). Effect of Weight-Bearing Activity on Foot Ulcer Incidence in People with Diabetic Peripheral Neuropathy: Feet First Randomized Controlled Trial. Phys. Ther.

[49] Tudor-Locke C, Bell RC, Myers AM, Harris SB, Ecclestone NA, Rodger NW, Lauzon N (2004). Controlled Outcome Evaluation of the First Step Program: A Daily Physical Activity Intervention for Individuals with Type II Diabetes. Int. J. Obes. Relat. Metab. Disord.

[50] Yates T, Davies M, Gorely T, Bull F, Khunti K (2008). Rationale, Design and Baseline Data from the Pre-Diabetes Risk Education and Physical Activity Recommendation and Encouragement (PREPARE) Programme Study: A Randomized Controlled Trial. Patient Educ. Couns.

[51] Bravata DM, Smith-Spangler C, Sundaram V, Gienger AL, Lin N, Lewis R, Stave CD, Olkin I, Sirard JR (2007). Using Pedometers to Increase Physical Activity and Improve Health: A Systematic Review. JAMA.

[52] Knols RH, de Bruin ED, Shirato K, Uebelhart D, Aaronson NK (2010). Physical Activity Interventions to Improve Daily Walking Activity in Cancer Survivors. BMC Cancer.

[53] Crouter SE, Schneider PL, Karabulut M, Bassett DR (2003). Validity of 10 Electronic Pedometers for Measuring Steps, Distance, and Energy Cost. Med. Sci. Sport. Exerc.

[54] Schneider PL, Crouter SE, Lukajic O, Bassett DR (2003). Accuracy and Reliability of 10 Pedometers for Measuring Steps over a 400-m Walk. Med. Sci. Sport. Exerc.

[55] Crouter SE, Schneider PL, Bassett DR (2005). Spring-Levered *versus* Piezo-Electric Pedometer Accuracy in Overweight and Obese Adults. Med. Sci. Sport. Exerc.

[56] Tudor-Locke CE, Bell RC, Myers AM, Harris SB, Lauzon N, Rodger NW (2002). Pedometer-Determined Ambulatory Activity in Individuals with Type 2 Diabetes. Diabetes Res. Clin. Pract.

[57] Corder K, Brage S, Ekelund U (2007). Accelerometers and Pedometers: Methodology and Clinical Application. Curr. Opin. Clin. Nutr. Metab. Care.

[58] Cyarto EV, Myers AM, Tudor-Locke C (2004). Pedometer Accuracy in Nursing Home and Community-Dwelling Older Adults. Med. Sci. Sport. Exerc.

[59] Pearson OR, Busse ME, van Deursen RW, Wiles CM (2004). Quantification of Walking Mobility in Neurological Disorders. QJM.

[60] Chen KY, Bassett DR (2005). The Technology of Accelerometry-Based Aactivity Monitors: Current and Future. Med. Sci. Sport. Exerc.

[61] Busse ME, Pearson OR, van Deursen R, Wiles CM (2004). Quantified Measurement of Activity Provides Insight into Motor Function and Recovery in Neurological Disease. J. Neurol. Neurosurg. Psychiat.

[62] Coleman KL, Smith DG, Boone DA, Joseph AW, del Aguila MA (1999). Step Activity Monitor: Long-Term, Continuous Recording of Ambulatory Function. J. Rehabil. Res. Dev.

[63] Duncan PW, Sullivan KJ, Behrman AL, Azen SP, Wu SS, Nadeau SE, Dobkin BH, Rose DK, Tilson JK (2007). Protocol for the Locomotor Experience Applied Post-stroke (LEAPS) Trial: A Randomized Controlled Trial. BMC Neurol.

[64] Shepherd EF, Toloza E, McClung CD, Schmalzried TP (1999). Step Activity Monitor: Increased Accuracy in Quantifying Ambulatory Activity. J. Orthop. Res.

[65] de Bruin ED, Najafi B, Murer K, Uebelhart D, Aminian K (2007). Quantification of Everyday Motor Function in a Geriatric Population. J. Rehabil. Res. Dev.

[66] Grant PM, Ryan CG, Tigbe WW, Granat MH (2006). The Validation of a Novel Activity Monitor in the Measurement of Posture and Motion during Everyday Activities. Br. J. Sport. Med.

[67] Ryan CG, Grant PM, Tigbe WW, Granat MH (2006). The Validity and Reliability of a Novel Activity Monitor as a Measure of Walking. Br. J Sport. Med.

[68] Brage S, Brage N, Ekelund U, Luan J, Franks PW, Froberg K, Wareham NJ (2006). Effect of Combined Movement and Heart Rate Monitor Placement on Physical Activity Estimates during Treadmill Locomotion and Free-Living. Eur. J. Appl. Physiol.

[69] Brage S, Brage N, Franks PW, Ekelund U, Wareham NJ (2005). Reliability and Validity of the Combined Heart Rate and Movement Sensor Actiheart. Eur. J. Clin. Nutr.

[70] Crouter SE, Churilla JR, Bassett DR (2008). Accuracy of the Actiheart for the Assessment of Energy Expenditure in Adults. Eur. J. Clin. Nutr.

[71] Zhang W, Moskowitz RW, Nuki G, Abramson S, Altman RD, Bierma-Zeinstra S, Arden N, Brandt KD, Croft P, Doherty M, Dougados M, Hochberg M, Hunter DJ, Kwoh K, Lohmander LS, Tugwell P (2007). OARSI Recommendations for the Management of Hip and Knee Osteoarthritis, Part I: Critical Appraisal of Existing Treatment Guidelines and Systematic Review of Current Research Evidence. Osteoarthritis Cartilage.

[72] Bosomworth NJ (2009). Exercise and Knee Osteoarthritis: Benefit or Hazard?. Can. Fam. Physician.

[73] Culhane KM, O'Connor M, Lyons D, Lyons GM (2005). Accelerometers in Rehabilitation Medicine for Older Adults. Age Ageing.

[74] Mathie MJ, Coster AC, Lovell NH, Celler BG (2004). Accelerometry: Providing an Integrated, Practical Method for Long-Term, Ambulatory Monitoring of Human Movement. Physiol. Meas.

[75] Steele BG, Belza B, Cain K, Warms C, Coppersmith J, Howard J (2003). Bodies in Motion: Monitoring Daily Activity and Exercise with Motion Sensors in People with Chronic Pulmonary Disease. J. Rehabil. Res. Dev.

[76] Berlin JE, Storti KL, Brach JS (2006). Using Activity Monitors to Measure Physical Activity in Free-Living Conditions. Phys. Ther.

[77] Tudor-Locke CE, Myers AM (2001). Challenges and Opportunities for Measuring Physical Activity in Sedentary Adults. Sports Med.

[78] Warms C (2006). Physical Activity Measurement in Persons with Chronic and Disabling Conditions: Methods, Strategies, and Issues. Fam. Community Health.

[79] Tudor-Locke C, Williams JE, Reis JP, Pluto D (2002). Utility of Pedometers for Assessing Physical Activity: Convergent Validity. Sports Med.

[80] Pitta F, Troosters T, Probst VS, Spruit MA, Decramer M, Gosselink R (2006). Quantifying Physical Activity in Daily Life with Questionnaires and Motion Sensors in COPD. Eur. Respir. J.

